# External quality assessment of cytomegalovirus DNA detection on dried blood spots

**DOI:** 10.1186/1471-2180-8-2

**Published:** 2008-01-08

**Authors:** Maria Barbi, William G MacKay, Sandro Binda, Anton M van Loon

**Affiliations:** 1 Department of Public Health-Microbiology-Virology, University of Milan, Italy; 2 The Neutral Office, Quality Control for Molecular Diagnostics, Glasgow, UK; 3 Department of Virology, University Medical Centre Utrecht, The Netherlands

## Abstract

**Background:**

Testing for viral DNA in neonatal blood dried on paper (DBS) has proved a valid means of diagnosing congenital CMV infection with both clinical and epidemiological relevance. To assess the quality of the detection of CMV-DNA on DBS in laboratories performing this test a proficiency panel consisting of nine samples with two blood spots on each filter paper was produced and distributed. Six samples were derived from whole blood, negative for CMV DNA and antibody, and spiked with cell-grown CMV Towne in various concentrations (7.3 × 10^2 ^– 9.6 × 10^5 ^copies/ml), one was a CMV positive clinical specimen (3.9 × 10^6 ^copies/ml), and two samples were CMV-negative whole blood.

**Results:**

The 27 responding laboratories from 14 countries submitted 33 datasets obtained by means of conventional PCR (n = 5) or real-time PCR (n = 28) technologies. A correct positive result was reported in at least 91% of datasets in samples with a viral load of 8.8 × 10^4 ^copies/ml or higher. However only 59% and 12% identified the 9.4 × 10^3 ^and 7.3 × 10^2 ^copies/ml samples, respectively, correctly as positive. False positive results were reported by 9% of laboratories and in 11% of datasets.

**Conclusion:**

These results indicate a clear need for improvement of methods as sensitivity and false-positivity still appear to be a major problem in a considerable number of laboratories.

## Background

Congenital CMV infection is the most widespread congenital infection in humans and is a major cause of neurological damages such as hearing loss, visual impairment and mental retardation in children. Diagnosis of congenital CMV infection requires laboratory testing done on samples collected in the first three weeks of life. Testing for CMV-DNA in neonatal blood collected on filter-paper (dried blood spot, DBS) has proved a valid means of diagnosis with both clinical and epidemiological relevance. Applications of this assay range from diagnosis in the neonatal period as an alternative to the conventional urine culture method to the unique quality of ascertaining whether damages arising during infancy are due to congenital infection [1,2].

Most laboratories use an in-house developed assay for detection of CMV-DNA in DBS. However, no international standard is available and previous external quality assessment studies have shown that the quality of nucleic acid amplification methods such as PCR varies considerably between laboratories [3,4]. Therefore a quality assessment study for the detection of CMV-DNA on DBS was recommended by the European Congenital CMV Initiative (ECCI) group and was organised by QCMD (Quality Control for Molecular Diagnostics). We report the results of the CMV-DNA amplification assays performed on a panel consisting of DBS with decreasing viral DNA content which was prepared for the study.

## Results and discussion

Thirty-three laboratories from 13 European countries and two from South Africa participated in the study. Twenty-seven laboratories submitted their results for a total of 33 datasets, as two respondents submitted two datasets and two sent three datasets each.

Few data on the characteristics of the participating laboratories are available as only a limited number of laboratories returned a completely filled out technical questionnaire. Thirteen of the 15 respondents operated in a University Hospital setting and 10 of the 15 were accredited. Five labs reported to have some experience in DBS testing. However, the annual number of specimens examined was usually small (<10–30 per year).

The molecular analysis was performed by means of in-house nested-PCR (n = 5) or real-time PCR (n = 28) technologies. Five datasets from three laboratories were obtained by means of commercial real-time PCR. Nucleic acid extraction was performed by means of commercial kits in 26 of 30 datasets. Only qualitative PCR results will be discussed as no more than five laboratories reported quantitative results of their real-time PCR assays.

Correct positive results on samples with a viral load of 8.8 × 10^4 ^copies/ml or higher were reported in ≥ 30/33 (90.7%) of datasets. However, performance on samples with lower viral loads dropped rapidly, with only 59% and 12% of correct positive results in samples with 9.4 × 10^3 ^and 7.3 × 10^2 ^copies/ml, respectively. Three laboratories reported a total of five false positive results (Table 1). The same three labs were among the six that reported positive results for the 7.3 × 10^2 ^copies/ml samples but one of them was unable to detect the 9.4 × 10^3 ^copies/ml samples.

**Table 1:** Composition of the panel and results of the pre-release testing and of the study

					**Correct qualitative results**			
					
					**PCR**			
					
		**Pre-release testing: qualitative results on DBS**			**Conventional**	**Real-time**		***Total results *** **n = 33 ** n(%)
			
**SAMPLE**	**Target sample concentration *(copies/ml)***	**MI**^1^	**UT1**^2^	**UT2**^2^	***In-house *n = 5 **n (%)	***Commercial *n = 5 **n (%)	***In-house *n = 23 **n (%)	
A	3.9 × 10^6^	+	+	+	5 (100.0)	5 (100.0)	23 (100.0)	33 (100.0)
B	9.6 × 10^5^	+	+	+	5 (100.0)	5 (100.0)	22 (95.7)	32 (97.0)
C	8.8 × 10^4^	+	+	+	5 (100.0)	5 (100.0)	20 (87.0)	30 (90.9)
D	9.4 × 10^3^	+	+	+	5 (100.0)	5 (100.0)	10 (43.5)	20 (60.6)
E	9.4 × 10^3^	+	-	+	5 (100.0)	5 (100.0)	9 (39.1)	19 (57.6)
F	7.3 × 10^2^	-	-	+	2 (40.0)	2 (40.0)	2 (8.7)	6 (18.2)
G	7.3 × 10^2^	-	-	-	0 (0.0)	1 (20.0)	1 (4.3)	2 (6.1)
H	negative	-	-	-	4 (80.0)	5 (100.0)	22 (95.7)	31 (93.9)
I	negative	-	-	-	4 (80.0)	4 (80.0)	22 (95.7)	30 (90.9)

^1 ^Nested PCR in-house on the Applied Biosystems (ABI) GeneAmp PCR System 9700. Sample pre-treatment: thermal shock (Binda *et al *2004). Analysis of one punch (3 mm) in triplicate.

^2 ^Real-time in house PCR on the Applied Biosystems (ABI) ABI PRISM 7900 Sequence Detection System. Sample pre-treatment: QIAGEN DSP kit. UT1: Analysis of three punches (3 mm each), UT2: Analysis of the entire dried blood spot.

Thus, the 9.4 × 10^3 ^copies/ml DNA concentration in the spiked whole blood represented the 50% sensitivity threshold of the tests.

The panel was designed primarily to assess sensitivity, as this was regarded as the overriding problem in the detection of CMV-DNA on dried blood spots. To allow comparison of the performance of laboratories, results were scored using a simple scoring system assigning two points for a correct result and zero points if the result was not correct. None of the datasets reported a maximum score of 18 points. On average, in house real-time PCR performed less well than conventional PCR and commercial real-time PCR. In fact the mean score attained by the former technology was 11.4 ± 2.7 versus 14.0 ± 1.4 and 14.8 ± 1.1 of the latter methods, respectively. These differences were statistically not significant.

The results of this first external quality assessment study indicate that methods should be improved in order to achieve better rates of sensitivity and specificity.

A previous study [5] showed that the median viral load in the cord blood of congenitally infected babies is 2.4 × 10^3 ^copies/ml. Thus our study indicates that over 50% of laboratories would not be able to identify congenitally infected babies by analysis of DBS. Only a few laboratories were able to detect a viral load of 7.3 × 10^2 ^copies/ml; the fact that half of these laboratories also reported false-positive results compromises the reliability of their results.

False positivity involved 9% of laboratories and 11% of datasets. This strongly affects the reliability of the retroactive congenital CMV diagnosis because of the impossibility of confirming the positive results through the classical CMV isolation assay on neonatal urine sample.

Lack of adherence to strict measures aimed at avoiding contamination and carry-over are the most probable cause of false positive results.

Testing CMV DNA on DBS presents several critical points. The methods of elution and extraction of DNA, the amount of spotted paper, the characteristics of the individual PCR tests and the criteria for positivity all can affect the performance of the test [2]. In this study we considered only the PCR methodology as assessment of the contribution of the various variables was not the intention of the study. Also, the number of laboratories using the same or similar protocols were too small for such an analysis.

## Conclusion

The importance of CMV-DNA detection on DBS relies on the peculiarity of the sample which is routinely collected in many countries and can be safely stored for years. In the field of congenital infection diagnosis and epidemiology it can be used to assess the prevalence of the infection in large population groups, to conduct neonatal screenings aimed at identifying and treating children at risk of permanent sequelae because of the infection, and to evaluate the burden of the infection as a cause of permanent damage.

The results of this first external quality assessment study indicate a clear need for improvement of methods. Future quality assessment programs should collect more detailed data on all the critical points of the assay. The results of such further quality assessment studies will aid laboratories in adjusting the assay in order to achieve better rates of sensitivity and specificity and to fully exploit the test.

## Methods

### Panel

Each participating laboratory received a panel consisting of nine samples with two blood spots on filter paper (903 Whatman) each (Table 1) and a questionnaire on the applied methods. One sample (A) was a CMV positive clinical specimen, six samples (B-G) were derived from whole blood, negative for CMV-DNA and antibody, and spiked with cell-grown CMV Towne diluted tenfold to various concentrations. The same CMV-negative whole blood was used for the negative controls (H-I). Both the CMV-negative blood and the clinical specimen were collected with EDTA as anti-coagulant. Each spot was prepared with 40 microlitres of sample. Materials were sent to laboratories in aluminium-coated envelopes by courier service at room temperature. Laboratories were given six weeks to return their results and the technical questionnaire to QCMD's Neutral Office in Glasgow.

### Pre-distribution testing

Samples were tested prior to dispensing on the cards. Target sample concentrations were determined by means of a commercial real-time PCR system (Nanogen Advanced Diagnostics srl Q-CMV Real-time Complete Kit) on the ABI 7300 Real-time PCR System (Applied Biosystem).

Pre-release testing was performed on the panel by two independent laboratories using nested or real-time in-house PCR assays (Table 1).

## Authors' contributions

MB conceived of the study, participated in the design of the study, drafted the manuscript. WGM elaborated the data and collaborated in drafting the manuscript. SB participated in the design of the study, prepared the panel and performed the preliminary assays. AMVL participated in the design of the study, coordinated the study and revised the manuscript. All authors read and approved the final manuscript.
